# The Evolving Role for Repeat Molecular Testing in Metastatic Colorectal Cancer

**DOI:** 10.3390/cancers18061007

**Published:** 2026-03-20

**Authors:** Nicholas D. Kendsersky, Mariah R. Erlick, Emerson Y. Chen, Hagen F. Kennecke

**Affiliations:** Knight Cancer Institute, Oregon Health & Science University, Portland, OR 97239, USA; kendsers@ohsu.edu (N.D.K.); erlick@ohsu.edu (M.R.E.);

**Keywords:** next-generation-sequencing, ctDNA, sequential NGS, metastatic colorectal cancer, precision medicine

## Abstract

Next-generation sequencing (NGS) has impacted the treatment landscape for mCRC, leading to improved outcomes through the use of molecularly targeted and immune checkpoint inhibitor therapies. The National Comprehensive Cancer Network (NCCN) and the American Society of Clinical Oncology (ASCO) recommend, at a minimum, initial testing to assess RAS, BRAF, HER2, and microsatellite instability (MSI)/mismatch repair (MMR) status, as these results determine therapeutic eligibility. Broader testing to identify the eligibility for tumor-agnostic therapy for a tumor mutation burden (TMB), NTRK gene fusions, and RET fusions is encouraged for all patients with advanced solid tumors. Patients with metastatic disease may develop progressive disease, often as a result of adaptive resistance mechanisms and selective therapeutic pressure on disease heterogeneity. Repeat biomarker testing at progression has the potential to define these resistance mechanisms and to guide the next therapy or clinical trial enrollment. While these practices have become more commonplace, unified guidelines have yet to be established. In this review of the literature, we evaluate the advantages and pitfalls of sequential biomarker testing during disease progression in patients with mCRC.

## Simple Summary

Molecular profiling is central to the management of metastatic colorectal cancer (mCRC), in which genomic alterations and biomarker expression guide prognosis and the selection of targeted therapies. Advances in next-generation sequencing, including blood-based circulating tumor DNA (ctDNA) assays, have enabled repeat molecular testing throughout the disease course to identify resistance mechanisms and newly actionable alterations. Despite increasing clinical adoption, guidance on when and how to perform sequential testing remains limited. This review summarizes the biological rationale for repeat molecular assessment in mCRC, compares available testing modalities, reviews biomarker-specific patterns of evolution, and highlights ongoing clinical trials that may inform future standards of care.

## 1. Introduction

CRC remains a significant global health burden and is the second leading cause of cancer-related death worldwide [1]. Over the past two decades, significant advances in our understanding of CRC genetics and tumor biology have fundamentally reshaped clinical management. Early models centered on the adenoma–carcinoma sequence have expanded to incorporate intratumoral heterogeneity, branched evolution, and the coexistence of both founder and progressor mutations [2,3]. These insights have informed the development of targeted therapies and immunotherapies, rendering molecular characterization a cornerstone of care in mCRC.

The treatment of mCRC is now fundamentally guided by a molecular genotype. Patients with RAS/BRAF wild-type, left-sided tumors are candidates for first-line EGFR inhibitor therapy with cetuximab or panitumumab in combination with chemotherapy, as established by the CALGB/SWOG 80405 and PARADIGM trials [4,5]. BRAFV600E-mutant disease, occurring in 8–12% of patients, confers poor prognosis and is now eligible for treatment with encorafenib-based combinations in the front-line setting [6,7]. HER2 overexpression or ERBB2 amplification, enriched in RAS/RAF wild-type tumors, may be treated with a dual HER2 blockade or an antibody–drug conjugate [8,9,10]. MSI-H/dMMR disease, accounting for 5% of mCRC cases, is associated with a dramatic response to the immune checkpoint blockade [11,12]. Beyond these established biomarkers, tumor-agnostic indications, including NTRK fusions, RET fusions, and TMB-high status, are recognized across solid tumors and apply to a subset of patients with mCRC [13,14,15]. The increasing complexity and number of genotype-guided treatment pairings underscore the notion that one single molecular test throughout a patient’s disease course may be inadequate. As patients progress through multiple lines of therapy, the genomic landscape evolves, and decisions that were appropriate at diagnosis may no longer reflect the biology defining resistant disease.

As therapeutic strategies have become increasingly genotype-directed, molecular testing platforms have also evolved. Testing has progressed from limited hotspot assays to broad, high-depth panels capable of detecting single-nucleotide variants, fusions, copy-number alterations, and tumor mutational burdens (TMB) [16,17]. Most recently, blood-based genomic assays, particularly ctDNA analysis, have emerged as a practical and minimally invasive complement or alternative to tissue sequencing [18,19]. CRC is particularly well-suited to blood-based genomic assays because of its relatively high ctDNA shedding rate compared with other epithelial malignancies, enabling real-time molecular surveillance with high sensitivity in many patients [20]. In parallel, targeted approaches such as digital droplet PCR (ddPCR) have emerged as highly sensitive tools for the detection and quantification of specific known variants, including RAS and BRAF mutations, and can detect mutant allele frequencies (MAF) below 0.1% [21,22].

Tissue biopsy remains the reference standard in several contexts. Histopathologic evaluation continues to confirm diagnosis, define histology, exclude dedifferentiation or neuroendocrine transformation, and provide architectural and immunophenotypic features that liquid biopsy cannot capture. Tissue-based NGS also remains valuable in cases of low ctDNA shedding, low tumor burden, or when required for trial eligibility [23,24,25].

Although the use of repeat tissue and blood NGS sampling has increased, there are limited guidelines and reviews of these practices, highlighting the urgent need for a critical appraisal [26]. Major guideline organizations, including the NCCN, the ASCO, and the ESMO, align on the importance of comprehensive molecular profiling at the time of metastatic diagnosis, recommending an assessment of RAS, BRAFV600E, HER2, and MSI/MMR status at a minimum, with broader panel testing encouraged to capture tumor-agnostic targets [27,28,29]. However, the guidance on sequential or repeat testing during disease progression remains limited and inconsistent. The NCCN advises against repeating molecular testing after the progression on cytotoxic chemotherapy but acknowledges that it may be appropriate after targeted therapy [27]. The ASCO similarly supports repeat testing after progression on targeted therapies; however, neither guideline specifies the platform, timing, or biomarker priorities [29]. The ESMO offers no specific recommendation on repeat testing at the time of progression. The absence of unified guidance creates a significant gap, which serves as the motivation for the present review. In this review, we evaluate the evidence for repeat molecular testing in mCRC, discuss biomarker-specific temporal changes, highlight scenarios in which repeat testing is clinically impactful, and outline ongoing trials that may inform future standards.

## 2. Materials and Methods

A narrative literature review was conducted to inform this perspective on sequential molecular testing in metastatic colorectal cancer. PubMed/MEDLINE was searched using predefined combinations of keywords that encompassed disease context, molecular profiling technologies, and biomarker-specific terms, including metastatic colorectal cancer, next-generation sequencing, repeat testing, tumor evolution, liquid biopsy, circulating tumor DNA, RAS, BRAF, HER, microsatellite instability, and tumor mutational burden. Relevant publications were selected based on clinical relevance, methodological quality, and contribution to understanding temporal genomic changes or testing strategies over the disease course. Additional studies were identified through reference list screening and expert knowledge in the field. As this manuscript is intended as a perspective rather than a systematic review or meta-analysis, the literature selection process was qualitative and hypothesis-generating to synthesize existing evidence and propose a conceptual framework for future investigations and clinical applications. As this work represents a narrative perspective rather than a systematic review, PRISMA reporting guidelines were not used.

## 3. Modalities for Repeat Testing and Current Clinical Standards

### 3.1. Current Recommendations in Genotyping Metastatic Colorectal Cancers

At the time of diagnosis of metastatic colorectal cancer, major guideline organizations (NCCN, ASCO, and ESMO) recommend comprehensive molecular profiling using NGS panels [27,28,29]. Both the NCCN and the ASCO prioritize tissue NGS over ctDNA, though blood-based assays are acceptable when tissue is unavailable or new biopsies are not feasible [27,29]. The ESMO recommends a routine use of tumor NGS in advanced colorectal cancer, with large multigene panels preferred if they add an acceptable extra cost compared with small panels, and recognizes that NGS can be performed on either tissue- or blood-based assays [28].

At disease progression in mCRC, the NCCN states that repeat molecular testing may not be necessary after progression on cytotoxic chemotherapy, as significant molecular changes are rarely observed, but may be considered after targeted therapies to guide future treatment decisions [27]. The ASCO similarly recommends that repeat genomic testing should be performed for patients with acquired resistance to targeted therapies, especially when known acquired resistance mechanisms may affect the choice of next-line therapy, and may also assist in identifying new targets after progression or prolonged stable disease on targeted therapies [29,30]. The ESMO does not provide specific guidance on repeat testing at progression.

A retrospective cohort study including 9134 patients across over 800 United States healthcare sites found that the receipt of targeted therapy in mCRC exceeded 50% irrespective of the RAS/RAF genotype [31]. The frequent use of targeted agents in mCRC, paired with the current guidelines recommending repeat molecular testing in patients progressing on targeted therapy, underscores the need for a comprehensive assessment of how the results may impact future care.

### 3.2. Options for Testing, Tissue vs. Blood

At the time of metastatic diagnosis or disease progression, clinicians must decide whether to pursue tissue re-biopsy or blood-based molecular testing. Formal guidelines do not explicitly address this decision, likely reflecting the assumption that key oncogenic drivers such as RAS arise early in tumorigenesis and are broadly represented across disease sites [3]. In practice, this decision is influenced by patient-specific factors, disease distribution, and prior testing history.

Blood-based assays offer several advantages, including minimal invasiveness, rapid turnaround time, and the potential to capture a composite genomic profile across multiple metastatic sites. In contrast, tissue biopsy preserves the histological context, allows for the evaluation of the tumor microenvironment, enables lesion-specific sampling, and may be necessary when ctDNA shedding is low.

**Figure 1:**
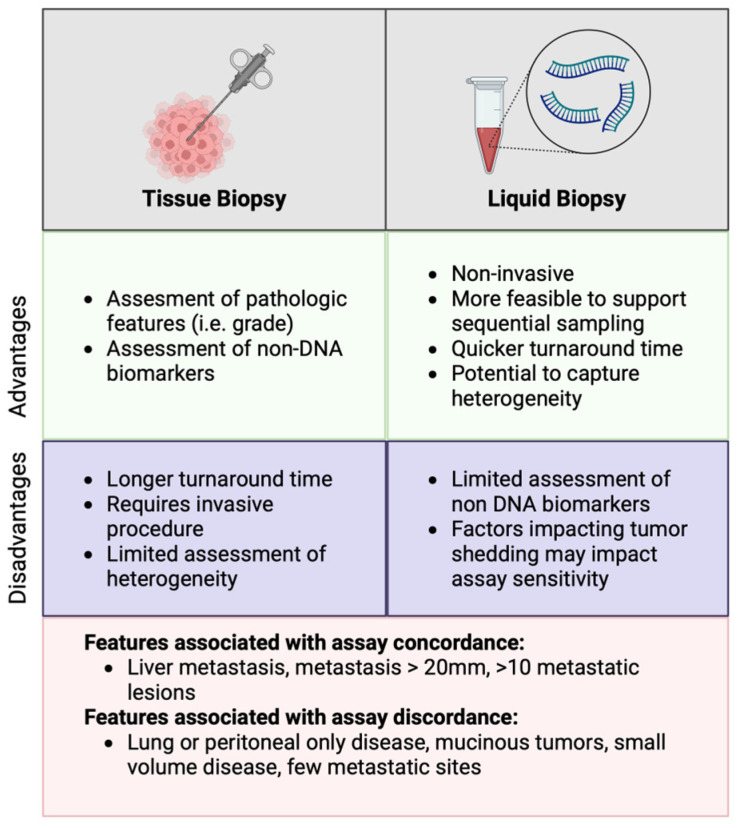
Advantages and disadvantages of tissue vs. liquid biopsy molecular testing are listed. Factors associated with assay concordance and discordance are described.

When ordered concurrently, tissue NGS and ctDNA may yield discordant results. A negative tissue result and a positive ctDNA result may occur due to tissue sampling heterogeneity, while a positive tissue and a negative ctDNA result often represent low ctDNA shedding. The factors leading to low shedding and discordance have been well described and include lung or peritoneal-only disease, small (<20 mm) metastatic lesion volume, few metastatic lesions (<10), and mucinous tumors [32,33,34]. The advantages and disadvantages of tissue and liquid biopsy, along with the factors impacting concordance, are summarized in Figure 1.

### 3.3. Diversity and Evolution of Assays

Commercial NGS panels differ in gene content, capture size, RNA integration, and biomarker outputs, and many have expanded over time [35,36,37]. For example, Tempus xT has expanded from an initial 595-gene panel to include over 640 genes with matched RNA sequencing, whereas Foundation Medicine uses a 324-gene panel [38,39,40]. The modern NGS platforms have expanded to include comprehensive RAS testing (beyond KRAS exon 2) and have been shown to identify 13% more patients with RAS mutations that are resistant to EGFR inhibition (EGFRi) [41]. A study of patients with advanced cancers (15% mCRC) compared a 46–50 gene hotspot assay to 409 gene whole exome assays, finding at least one new actionable gene alteration in 214/521 patients, leading to matched therapy in 19% of these cases with an associated improvement in overall survival compared to non-matched treatments [42]. Although the panel design varies across laboratories, large-scale concordance studies have demonstrated that the agreement in variant detection approaches 95% across widely used NGS platforms [43]. Because overall survival in mCRC is measured in years, the initial testing in some patients may have been limited to hotspot analysis. Therefore, repeat testing with a comprehensive panel in this setting would be justified.

Tumor mutational burden (TMB) warrants additional consideration. The studies across various tumor types demonstrate that liquid biopsy TMB values are often higher than those obtained from tissue sampling, frequently exceeding meaningful clinical thresholds, such as the 10 mut/Mb cutoff used to guide immunotherapy [44,45]. An extensive cross-platform analysis by Sturgill et al. reported that the median TMB estimates were higher in ctDNA than in solid-tissue profiling, independent of cancer type, and that more than one-third of cases were classified as having a conflicting TMB high and low status [45]. In addition, this study highlighted the differences in blood TMB distributions across vendors (Foundation Medicine mean TMB = 3.8 mut/Mb; Guardant Health mean TMB = 10.5 mut/Mb) [45].

Liquid biopsy technologies have evolved considerably and vary in technique. Tumor-agnostic platforms (e.g., Guardant360, FoundationOne Liquid CDx, and Tempus xF+) utilize hybrid-capture NGS to quantify ctDNA without prior knowledge of a patient’s tumor genotype, enabling a flexible application in metastatic settings. While tumor-informed assays that leverage personalized mutation signatures derived from prior tissue biopsies have demonstrated strong utility in detecting early recurrence, their role in the metastatic setting remains uncertain [46,47]. Beyond NGS-based platforms, digital droplet PCR (ddPCR) represents an alternative liquid biopsy approach with distinct technical characteristics. Rather than broadly profiling the genome, ddPCR partitions a DNA sample into thousands of individual droplets to detect pre-specified variants with very high analytical sensitivity that are capable of identifying mutant allele frequencies (MAF) below 0.1% [21,22]. The application of ddPCR has been limited to research settings to track MAF over the treatment course and is not universally available as a commercial assay [48,49].

### 3.4. Heterogeneity and Testing Discordance

Molecular testing at the time of diagnosis and when repeated is subject to inter- and intra-tumoral heterogeneity and disease-specific factors such as metastatic organ site, metastasis size, and quantity. This heterogeneity has been captured by parallel efforts to sequence both primary tumor and metastatic sites, leading to discordant rates of 3–10% in RAS variants, 5–8% in BRAF, and 7–10% in PIK3CA [50,51]. The discordance rates of HER2 overexpression have been reported across gastrointestinal malignancies and in CRC, and they may approach 10–27% when comparing primary tumors to lymph node or liver metastasis [52,53]. Several studies have compared TMB at the primary and metastatic sites. One extensive, unmatched pan-cancer study demonstrated a modest increase in TMB across cohorts of primary and metastatic disease sites, whereas another smaller study found TMB to be 36% higher in matched metastatic biopsy sites than in primary tumors [54,55]. These discrepancies may arise from sampling biases, intratumoral heterogeneity, or the emergence of subclonal metastasis-specific variants that are not represented in the primary lesion.

The impact of discordance on clinical outcomes has been explored. In one small study, patients with matched RAS genotyping from primary and metastatic liver sites revealed that patients with a wild-type primary tumor and RAS-mutated liver resection (n = 10, 9.4% of patients) were a strong predictor of poor overall survival, while the converse (RAS mutant primary tumor/RAS WT metastasis) was not associated with a worsened survival [56].

Discordance is observed in patients undergoing simultaneous liquid and tissue biopsies. It can be attributed to low shedding in the tissue-positive/blood-negative results or intertumoral heterogeneity in the tissue-negative/blood-positive results. One large cohort study evaluating concurrent blood and tissue NGS in 3209 patients with metastatic solid tumors identified actionable mutations in 45.1% of the patients, with a 66.4% concordance between the assays; discordant actionable mutations were detected in 9.3% and 24.2% of the liquid and tissue assays, respectively [32]. Of the 923 mCRC patients in this study, a 74.5% concordance in actionable mutations between the two assays was observed, with ctDNA identifying unique actionable alterations in 6.3% (n = 39) of the patients and tissue-based NGS in 19.2% (n = 119) of the patients [32]. Specific biomarker discordances have been reported, ranging from 10 to 36% in RAS variants, 10–25% in BRAF V600E, 30–60% in HER2 amplification, 13–32% in MSI status, and 13–28% in PIK3CA [23,32,33,48,50,51,57,58,59,60,61,62]. A wide range of reported discordance between tissue and blood-based assays reflects the dependence of blood assays on increased shedding and assay variation.

Outside of the clinical trials, concurrent tissue and blood NGS are rarely performed; however, the therapeutic implications of platform discordance are clinically meaningful and warrant consideration. Before interpreting discordant results, clinicians should recognize that ctDNA shedding is not uniform across patients and that disease characteristics strongly predict the performance of liquid biopsies. In low shedding contexts, a tissue biopsy is likely to perform better and should be preferred when it is feasible.

With this clinical framework in mind, the implications of discordance are biomarker-specific. For the RAS variants, the operative principle is that a mutation detected by either assay is sufficient to preclude EGFRi therapy: patients with tissue RAS wild-type tumors but ctDNA RAS mutations have significantly shorter PFS when treated with anti-EGFR therapy compared with concordant wild-type patients [63,64]. For BRAF V600E, the same assay-agnostic approach applies, and treatment with BRAF inhibitors may be rationalized based on the tissue or blood mutation status. High discordant rates in HER2 amplification detection reflect challenges in detecting copy number variations in plasma, and discordant results affect treatment selection. The amplification detected in blood without confirmatory tissue IHC would qualify patients for dual HER2-targeted therapy, whereas IHC 3+ is required for eligibility to trastuzumab deruxtecan [8,27,65]. The discordance in MSI is largely driven by ctDNA-negative tissue-positive results, maintaining tissue as the optimal assay [32,59]. A positive MSI-H ctDNA result with a negative tissue biopsy may warrant a repeat tissue biopsy. PIK3CA mutations are not actionable biomarkers in mCRC, though they may predict resistance to other targeted therapies [60,66]. Additionally, while aspirin may improve the recurrence-free survival in the adjuvant setting of PIK3CA-mutated CRC, it remains to be established whether it confers any benefit in metastatic disease [67]. A summary of discordance between the primary and metastatic tissue biopsy, along with tissue and liquid biopsy, is provided in Table 1.

Other markers have not yet had any data describing the discordance between ctDNA and the tissue. NTRK fusions fuse the encoded tyrosine kinase gene to a partner gene, leading to constitutive activation of TRK. NTRK fusions are relatively rare, occurring in approximately 0.7% of all CRCs, though they can be as high as 7% in the MSI-high CRC subset [68]. This fusion leads to a remarkable sensitivity to TRK inhibitors (entrectinib and larotrectinib), which are otherwise not first-line agents for CRC [69,70]. There is no direct research on the concordance between ctDNA and the primary or metastatic tumor sites. Similarly, NRG1 is a ligand in the ERG family that fuses with partner genes, leading to the downstream constitutive activation of tyrosine kinases. These mutations occur in approximately 0.17% of CRC cases [71]. There is no data on concordance between ctDNA and the tumor sites; however, one study examined the transcriptomes of primary and metastatic sites and found that NRG mutations were present (or absent) with a 100% concordance [70].

### 3.5. Impact of Therapy Exposure on Sequential Testing

The characterization of molecular changes following the delivery of targeted therapy has been a research focus since the introduction of EGFRi into the mCRC treatment landscape. These efforts suggest that the acquired resistance to EGFR-targeted therapy occurs via two main mechanisms: emergence of resistant clones harboring activating mutations in downstream effectors of the EGFR pathway, such as RAS, RAF, and MEK, or mutations in the EGFR extracellular domain that impair antibody binding to the receptor [72,73,74]. The dynamic nature of clonal resistance has been further demonstrated, showing that the discontinuation of EGFR-targeted therapy often results in the decay of these resistant clones with a half-life as short as 4.4 months [73,75,76]. The pattern of the initial clonal expansion of resistant variants during therapy, followed by contraction upon treatment withdrawal as sensitive clones regain a fitness advantage, is a phenomenon directly exploited by EGFRi rechallenge strategies [77,78,79]. Parallel efforts in patients receiving BRAF V600E-targeted therapies have revealed mechanisms of resistance that rely upon the restoration of MAPK pathway signaling through RAF dimerization, KRAS amplification, or MEK mutations [80].

In addition to providing a rationale for treatment discontinuation in the setting of resistance mechanisms, sequential testing may uncover new targetable findings or eligibility for clinical trials. Repeat testing at the time of progression has gained traction in various tumor types, including lung, prostate, and breast cancers [81,82]. In mCRC, longitudinal circulating tumor DNA (ctDNA) profiling is emerging as a practical tool for this purpose. In one series of 501 patients, sequential ctDNA analysis identified new genomic alterations in 71% of cases, including single-nucleotide variants, amplifications, and microsatellite instability [83].

## 4. Implications of Repeat Genotyping at Progression in Practice

### 4.1. Longitudinal RAS Monitoring

The RAS variants are the most frequently mutated oncogenes in mCRC and are estimated to occur in half of the patients diagnosed with mCRC [84]. The current guidelines from the NCCN, the ESMO, and the ASCO are aligned in emphasizing the importance of RAS sequencing in mCRC, as wild-type RAS status is required when considering EGFRi in first-line therapy for left-sided mCRC [27,28,29]. This is because mutant RAS activates downstream signaling in the EGFR pathway and confers resistance to EGFRi. The landmark CALGB/SWOG 80405 trial (NCT00265850) supports this approach, demonstrating an improved overall survival with first-line EGFRi plus chemotherapy in patients with left-sided, RAS-wild-type mCRC, thereby establishing this regimen as a preferred option in this subgroup [5].

The mutations in the EGFR/RAS pathway that were not present at the time of diagnosis may be present on sequential NGS. The patients receiving EGFRi with available pre- and post-treatment molecular testing have demonstrated acquired mutations in KRAS, NRAS, BRAF, MET, and EGFR, among others [73,85]. An analysis of patients treated with Panitumumab + mFOLFOX6 in the PARADIGM study revealed at least one new genetic alteration in 69% of patients [86]. Furthermore, patients with alterations in the RTK/RAS axis had significantly shorter post-progression survival (PPS) than those without acquired alterations, 13.2 vs. 18.8 months [86].

For patients who remain RAS-WT at the time of progression, the utility of EGFRi rechallenge has been of clinical interest. Initially, small studies such as CHRONOS, CAVE, and CRICKET suggested a clinical benefit with this rechallenge approach [77,78,87]. The large phase III FIRE-4 trial (NCT02934529) evaluated rechallenge in patients who remained RAS-WT (by tissue or liquid biopsy) at the second progression after EGFRi free treatment interval, showing that, while cetuximab rechallenge improved radiographic response rate compared with the physician’s choice (28.9% vs. 11.9%), the trial did not meet its primary OS endpoint (17.6 vs. 15.1 months) [79]. These results suggest that RAS clearance alone may not be a complete biomarker of rechallenge sensitivity.

In contrast to emergent RAS mutations, a subset of patients initially harboring RAS-mutant disease may develop a RAS WT ctDNA profile after cytotoxic therapy, potentially restoring EGFR pathway dependency and raising the possibility of seeing benefits from EGFRi [88]. The trials to determine the utility of EGFR-targeted therapy in this patient population are ongoing [89]. Several additional trials utilizing ctDNA to guide treatment selection and prognosis are also ongoing. LIBImAb (NCT04776655) investigates bevacizumab versus cetuximab in combination with FOLFIRI in untreated patients with RAS/BRAF WT mCRC on tumor tissue and RAS/BRAF mutations detected through ctDNA analysis [90]. TACT-D (NCT03844620) and RAPID-1 (NCT04786600) aim to establish a prognostic role for sequential ctDNA and to provide a rationale for early switch of therapy before radiographic progression [91,92].

### 4.2. HER2 Biomarker Testing

The amplification of ERBB2 and/or HER2 protein overexpression by immunohistochemistry (IHC) occurs in approximately 3% of unselected mCRC and is enriched among RAS/BRAF wild-type tumors, where the prevalence approaches 8–10% [93]. The NCCN, the ESMO, and the ASCO all recommend molecular testing in mCRC that are RAS/BRAF WT to identify the patients who may benefit from HER2-directed therapy [27,28,29]. Currently, HER2-targeted strategies, including trastuzumab/pertuzumab, tucatinib-trastuzumab, and trastuzumab-deruxtecan, are primarily used after progression on standard cytotoxic chemotherapy. However, ongoing trials such as MOUNTAINEER-3 (NCT05253651) are evaluating the impact of first-line HER2 therapy on survival [94].

HER2 testing in CRC is most commonly initiated on tissue using IHC and is reported as 0/1+/2+/3+ based on membranous staining intensity and extent [95]. The variations in HER2 staining patterns compared to other cancer types have led to CRC-specific scoring systems that emphasize strong membranous staining in a substantial fraction of tumor cells to define IHC 3+ [8,93]. In equivocal cases such as HER2 2+ by IHC, ERBB2 amplification is assessed using in situ hybridization (ISH), and, in parallel, NGS panels may frequently report ERBB2 copy-number gains [93]. The final consensus criteria proposed in the HERCALES trial (NCT03225937) require 3+ membranous HER2 staining in ≥50% of cells or confirmed amplification in the event of equivocal staining as defined by 3+ membranous staining in >10% but <50% of cells or 2+ membranous staining in ≥50% of cells [8].

The significant variability and discordance observed in the HER2 expression, along with the non-uniform definitions for HER2 positivity used in pivotal clinical trials, have complicated the treatment landscape for HER2-positive mCRC. DESTINY CR01 (NCT03384940) enrolled three cohorts, including Cohort A]HER2 3+ or HER2 2+ with confirmatory FISH, Cohort B] HER2 2+ with negative FISH, and Cohort 3] HER2 1+ (negative FISH) [10]. DESTINY CR02 (NCT04744831) employed stricter criteria, omitting patients meeting criteria for cohorts two and three in DESTINY CR01, whereas DESTINY PanTUMOR02 (NCT04482309) enrolled patients with HER2 3+/2+ by local or central testing [9,65]. The eligibility criteria for the MOUNTAINEER trial include patients with 3+ by IHC, 2+ by IHC with amplification by FISH or chromogenic in situ hybridization (CISH), or amplification by NGS [94].

While mechanisms of resistance to HER2-directed therapy remain incompletely understood in CRC, repeat molecular testing at the time of progression has demonstrated alterations in HER2, EGFR, PIK3CA, and PTEN, representing similar escape mechanisms to those seen in breast cancer [96,97]. The loss of HER2 expression likely represents another mechanism of resistance, as observed in other gastrointestinal malignancies, occurring in 14 to 60% of cases with evaluated pre- and post-treatment biopsies [98,99,100]. HER2 overexpression or ERBB2 amplification, not detected on initial testing, may be identified on repeat molecular testing after non-HER2-targeted therapies, such as EGFRi [85,101]. The benefit of HER2-targeted therapies in this specific context is poorly characterized. TRIUMPH (NCT02091141), a small Phase II study, demonstrated that ctDNA genotyping can identify patients who would benefit from dual HER2 blockade, and post hoc analysis showed that serial measurements may help monitor treatment response [102].

### 4.3. BRAF Testing

Between 8% and 12% of the patients diagnosed with mCRC harbor the BRAFV600E mutation. To guide treatment decisions, the NCCN, the ESMO, and the ASCO recommend or advise BRAF molecular testing at the time of mCRC diagnosis [27,28,29]. The testing guides both therapeutic selection and prognosis, as patients with BRAF-mutated mCRC have been shown to have worse outcomes than those with BRAF-WT mCRC [103]. Based on the BEACON and BREAKWATER studies, patients with BRAFV600E-mutant mCRC are candidates for targeted therapy with encorafenib plus anti-EGFR, which is increasingly used earlier in the treatment course [6,7].

Despite these therapeutic advances, outcomes for BRAF-mutated mCRC remain suboptimal, and mechanisms of resistance to these treatments have been well described. Sequential molecular studies at the time of progression have identified new alterations, including MET and BRAF amplifications, as well as KRAS/NRAS, MAP2K1, and PIK3R1 mutations [104,105]. With the presence of KRAS/NRAS or MAP2K1 mutations, further BRAF-directed therapy (e.g., adding binimetinib to cetuximab/encorafenib) or switching to other BRAF/MEK inhibitors is likely ineffective [106]. While rare, repeat testing may reveal new BRAF alterations in previously WT patients, but the prognostic and therapeutic implications of non-V600E mutations remain unestablished [107].

In patients with BRAF V600E, serial liquid biopsies may inform prognosis and treatment response via a surrogate marker of allele frequency. In a translational analysis of the FIRE-4.5 trial, patients with tissue molecular testing confirming BRAF V600E had improved PFS and OS when liquid biopsy at baseline or after treatment was BRAF WT [108]. Concordantly, patients with higher ctDNA BRAF V600E MAF had worse PFS and OS than those with lower MAF [108]. As BRAF-targeted therapies enter the frontline setting for BRAF V600E-mutated mCRC, repeat testing in blood or tumor tissue may provide insights into the prognosis and potential rechallenge at progression.

### 4.4. Longitudinal TMB

The tumor mutational burden (TMB), calculated as the total number of somatic variants per defined region of the tumor genome, is now routinely reported across NGS platforms as a biomarker for immunotherapy response. This has been driven in part by the results of KEYNOTE-158, which demonstrated a clinical benefit with pembrolizumab in patients with non-colorectal-advanced cancers and TMB-high tumors (i.e., TMB > 10) [13]. It is hypothesized that higher TMB leads to an increased expression of high-quality neoantigens, which promotes T-cell reactivity and improved responses to immunotherapy [109,110]. In patients with mCRC, a TMB-high genotype is almost exclusively observed in patients with a deficient mismatch repair (dMMR) or microsatellite instability (MSI) disease; however, a TMB-high genotype can also be observed in mismatch repair-proficient (pMMR) and microsatellite-stable (MSS) patients. The molecular profiling of these unique cases has revealed alterations in proofreading polymerases (POLE and POLD1), DNA damage repair (DDR), and mutations in epigenetic modifiers [111].

While immunotherapy has dramatically improved outcomes in MSI/dMMR/POLD1-mutant mCRC, it has not produced the same benefit in patients with TMB-high MSS/pMMR disease [112,113]. Efforts to identify a subgroup of MSS patients who may benefit from immunotherapy have been a focus of research. The ATEZOTRIBE study, which compared upfront FOLFOXIRI plus Bevacizumab with and without Atezolizumab, did not show a significant difference in the overall survival in the intention-to-treat population; however, the patients with a high Immunocore (density of CD8+ and PD-L1+ cells) and a high TMB derived benefits from the addition of immunotherapy [114]. The analysis of the CO.26 study, which randomized heavily pretreated MSS/pMMR mCRC to a combination of durvalumab + tremelimumab or best supportive care, showed that patients with elevated blood TMB may benefit from immunotherapy, while TMB-high tissue did not maintain this benefit [115,116,117].

It has been speculated that, over time and under therapeutic pressure, TMB may not be a static measure. However, in mCRC, conflicting information regarding TMB trends has emerged in the literature: some studies report stable TMB over time and under therapeutic pressure, whereas others report a general increase in TMB [118,119]. Heterogeneity and discordance between blood-based and tissue TMB further complicate this issue, leaving the threshold for considering a trial of immunotherapy unclear. Whether acquired TMB-high may serve as a biomarker of response to immunotherapy has been investigated. One recent small retrospective study evaluated MSS/pMMR patients for acquired TMB after progression on targeted therapy (n = 9) and found a greater increase in blood TMB than in tissue TMB [120]. Three of these patients received immunotherapy; however, no patients responded, and further molecular evaluation revealed increased subclonal alterations in the blood-based assay and limited tumor-infiltrating lymphocytes or PD-L1 expression in the tissue [120]. Larger studies are needed to fully characterize the benefit, or lack thereof, of immunotherapy in patients with mCRC and acquired TMB-high.

### 4.5. Repeat Genotyping in Clinical Practice

While reviews of nationwide databases demonstrate strong trends toward an increasing proportion of patients undergoing multiple NGS tests, and repeat NGS has become the standard in select cancer types at the time of progression, no consensus guideline exists for mCRC [26]. In a 2022 ASCO Provisional Opinion, irrespective of primary cancer type, repeat testing was rationalized for the patients with limited-coverage NGS panels that were previously performed, for patients who progressed on prior targeted therapies, and for cases in which resistance mechanisms would affect subsequent line treatment [30]. However, this recommendation does not consider liquid versus tissue testing. In clinical trials, many studies have adopted a hybrid approach to sequencing, whereby initial molecular testing is performed on the tissue, and subsequent analysis is conducted on the blood. The anticipated findings upon repeat testing and clinical implications are summarized in Table 2.

## 5. Discussion

The expanding role of molecular profiling in mCRC has introduced new opportunities regarding the clinical utility of sequential genomic testing. While baseline molecular characterization is firmly established as a prerequisite for current mCRC management, the evidence reviewed here underscores the dynamic tumor biology over the course of the treatment. Intratumoral heterogeneity, selective pressure from systemic therapy, and assay-related limitations can all contribute to clinically meaningful differences between baseline and progression molecular profiles. Consequently, repeat testing may identify actionable alterations, resistance mechanisms, or prognostic signals that are not apparent at the time of diagnosis.

The clinical data support the premise that broader, longitudinal molecular interrogation can influence treatment decisions, particularly when the initial testing was confined to limited hotspot assays [42]. Beyond panel breadth, longitudinal testing has shown that new genomic alterations frequently emerge over time, particularly following exposure to targeted therapy, affecting key pathways such as RAS, HER2, BRAF, and DNA damage repair. Importantly, repeated profiling may also reveal the persistence of resistant clones or loss of target expression, information that can be equally valuable for avoiding ineffective therapies.

The trials in precision oncology have further informed this evolving paradigm. The ROME trial (NCT04591431), which mandated repeat tumor or plasma profiling across advanced solid tumors, including CRC, demonstrated improved response rates among patients receiving biomarker-matched therapy compared with standard-of-care treatment, providing proof of concept that evolution-informed treatment strategies can yield clinically meaningful benefits [121]. ROME supports the feasibility and potential relevance of repeat molecular testing to guide therapy selection after disease progression. The additional pan-cancer efforts, such as NCI-MATCH (NCT02465060) and the ongoing COMBO-MATCH trial (NCT05564377), reinforce this concept, with COMBO-MATCH representing a next step by explicitly targeting resistance mechanisms through rational combination strategies informed by acquired genomic alterations [122,123].

The emergence of novel biomarkers is folded into these recent and ongoing trials. Pan-cancer efforts such as NCI-MATCH, recently concluded in 2023, and NCI-ComboMATCH, with ongoing enrollment, use a tumor-agnostic approach that can rapidly identify patients with known and novel biomarkers. The paradigm shift toward treating based on mutation, rather than cancer type, also demonstrated that a single NGS assay can effectively and efficiently identify patients who may benefit from targeted therapies; NCI-MATCH also quantified the patients who may benefit from targeted therapies at a notable 38%. NCI-ComboMATCH further expanded on this premise by identifying genomically directed combination therapies. The efficient identification of not only novel biomarkers but also the patients who may benefit from them remains an ongoing research topic.

**Figure 2:**
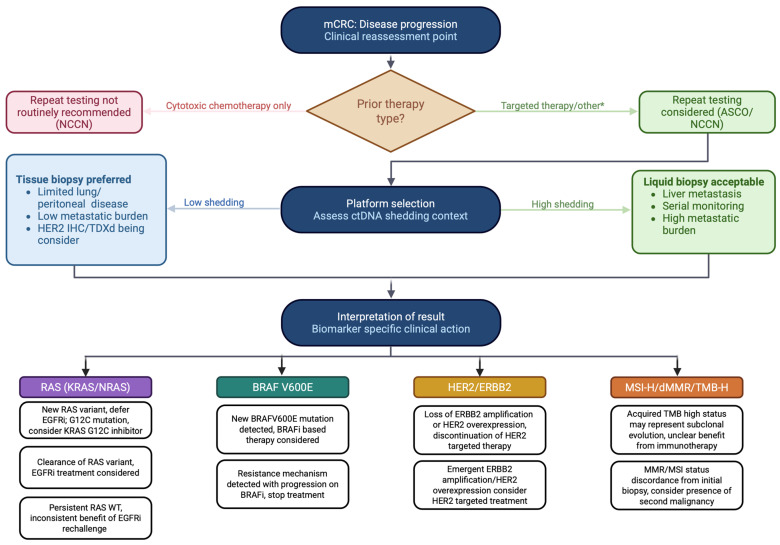
Clinical algorithm for repeat molecular testing in metastatic colorectal cancer at the time of disease progression * In addition to prior targeted treatment, other indications for repeat testing include prior limited gene panel and to determine clinical trial eligibility.

While these precision medicine-focused efforts generate enthusiasm, everyday clinical application requires careful interpretation. The results must be contextualized by platform, disease distribution, and patterns of assay discordance—particularly the false-negative risk of liquid biopsy in low shedding contexts. Conversely, a resistance variant identified on either platform carries therapeutic significance and should not be dismissed based on discordant negative results from the other modality. To consolidate the evidence reviewed here into a practical framework, we propose a stepwise clinical algorithm for repeat molecular testing in mCRC at disease progression (Figure 2), structured around three sequential decisions: whether testing is indicated, which platform is appropriate given the metastatic distribution and expected ctDNA shedding, and how biomarker-specific findings should direct next-line therapy. Ultimately, repeat molecular testing in mCRC is a context-dependent decision and is most impactful when performed after progression on targeted therapy, when results may inform the next line of therapy.

This perspective review has several limitations. The literature review was narrative and non-systematic, precluding formal assessment of bias or quantitative synthesis. The studies discussed are heterogeneous in terms of sequencing platforms, timing of molecular testing, and clinical context, limiting direct comparability. Moreover, much of the available evidence is retrospective or exploratory, with relatively few prospective trials specifically designed to evaluate the clinical utility of sequential molecular testing. Given the rapid evolution of molecular diagnostics and targeted therapies, some conclusions may require refinement as new data emerge. Finally, our discussion is limited to the biological and treatment rationale for repeat testing, omitting patient perspectives, access to repeat testing, and insurance coverage, which represent meaningful directions for future work.

## 6. Conclusions

Repeat molecular testing has an established role in the management of patients with advanced colorectal cancer and may be considered after progression on targeted therapy, in cases of mixed response, when HER2-directed therapy is being considered, to guide re-treatment decisions, and when trial eligibility is being assessed. Outside of these indications, the role for repeat or longitudinal molecular testing remains experimental and evolving.

## Figures and Tables

**Table 1:** The summary of biomarker concordance across tissue metastasis and blood assay.

Biomarker	Primary vs. Met Discordance	Tissue vs. ctDNA Discordance	Clinical Implication
RAS Variants	3–10% [50,51,56] *	10–36% [23,32,33,57]	• Tissue or ctDNA with RAS/RAF mutations predicts poor response to EGFRi. • Adagrasib/Sotorasib (FDA-approved 2nd line) may be considered for second-line treatment in KRAS G12C mutants.
BRAFV600E	5–8% [50,51] *	10–25% [32,33,48]	• Historically associated with poorer prognosis. • Patients with tissue or ctDNA positivity are eligible for Encorafenib + Cetuximab + FOLFOX/FOLFIRI (FDA-approved 1st line).
HER2	10–27% [52,53]	30–60% [32,58]	• Heterogeneity is common; high rates of discordance may increase rates of false-negative testing. • HER2 overexpressing (IHC 3+) can receive TDXd; ERBB2 amplified to consider dual HER2-targeted therapy.
MSI-H	<2% [62]	13–32% [32,59] **	• High concordance across tissue sites; discordance may suggest secondary malignancy. • Checkpoint inhibitor therapy is FDA-approved in the front-line setting for patients with MSI-H.
PIK3CA	7–10% [51] *	13–28% [60,61]	• Adjuvant low-dose aspirin has demonstrated decreased risk of recurrence in non-metastatic CRC. • PIK3CA is implicated in EGFRi therapy resistance and is a selective biomarker for clinical trials.

Legend: The pooled frequency of genotype discordance between the primary and metastatic tissue biopsy is listed. The pooled frequency of genotype discordance between the tissue-based and blood-based NGS is listed. The clinical implications of discordance are described. * Data derived from systematic review/meta-analysis. ** Data pooled from multiple solid tumors.

**Table 2:** The potential findings at the time of repeat molecular testing.

Initial Genotype	Findings on Repeat Testing	Mechanism of Change	Clinical Action
RAS mutant	RAS mutant	Persistent clone	Continue to defer EGFRi.
	RAS WT	Clonal selection	Ongoing trials to determine the benefit of EGFRi in this setting.
RAS WT	Acquired RAS mutant	Clonal selection	Defer EGFRi Rechallenge.
	Persistent RAS WT	Persistent clone	Unclear benefit with RAS rechallenge.
HER2 overexpression/ERBB2 amp	Loss of ERBB2 amp/expression	Clonal selection	Discontinuation of HER2-targeted therapy.
	Persistence of HER2 overexpression	Persistent clone	Consider trastuzumab-deruxtecan in IHC 3+.
BRAFV600E	Increased/decreased MAF	Treatment effect	Discontinue upon progression/continue treatment.
	Emergent mechanisms of resistance	Clonal selection	Discontinue treatment upon progression.
TMB	Acquired TMB high	Subclonal evolution	Increased TMB may not lead to a response to immunotherapy.
	Stable	N/A	No strong rationale for immunotherapy.

Legend: The initial genotype with anticipated findings after progression of disease are reported. The mechanism of these changes is listed along with potential clinical action to follow.

## Data Availability

No new data were created or analyzed in this study. Data sharing is not applicable to this article.
